# A Critical Review on Pulsed Electric Field: A Novel Technology for the Extraction of Phytoconstituents

**DOI:** 10.3390/molecules26164893

**Published:** 2021-08-12

**Authors:** Muhammad Modassar A. N. Ranjha, Rabia Kanwal, Bakhtawar Shafique, Rai Naveed Arshad, Shafeeqa Irfan, Marek Kieliszek, Przemysław Łukasz Kowalczewski, Muhammad Irfan, Muhammad Zubair Khalid, Ume Roobab, Rana Muhammad Aadil

**Affiliations:** 1 Institute of Food Science and Nutrition, University of Sargodha, Sargodha 40100, Pakistan; modassarranjha@gmail.com (M.M.A.N.R.); kanwalrabia043@gmail.com (R.K.); bakhtawarShafique111@gmail.com (B.S.); shafeeqairfan@gmail.com (S.I.); 2 Institute of High Voltage and High Current, Faculty of Engineering, Universiti Teknologi Malaysia, Skudai 81310, Johor, Malaysia; rainaveed77@gmail.com; 3 Department of Food Biotechnology and Microbiology, Institute of Food Sciences, Warsaw University of Life Sciences—SGGW, Nowoursynowska 159 C, 02-776 Warsaw, Poland; 4 Department of Food Technology of Plant Origin, Poznań University of Life Sciences, 31 Wojska Polskiego St., 60-624 Poznań, Poland; przemyslaw.kowalczewski@up.poznan.pl; 5 Department of Food Engineering, University of Agriculture, Faisalabad 38000, Pakistan; ikhan7791@outlook.com; 6 Department of Food Science, Government College University, Faisalabad 38000, Pakistan; zubairkhalid730@gmail.com; 7 School of Food Science and Engineering, South China University of Technology, Guangzhou 510006, China; mahroba73@gmail.com; 8 National Institute of Food Science and Technology, University of Agriculture Faisalabad, Faisalabad 38000, Pakistan

**Keywords:** PEF, green extraction techniques, phytochemical extraction, oil extraction, food waste

## Abstract

Different parts of a plant (seeds, fruits, flower, leaves, stem, and roots) contain numerous biologically active compounds called “phytoconstituents” that consist of phenolics, minerals, amino acids, and vitamins. The conventional techniques applied to extract these phytoconstituents have several drawbacks including poor performance, low yields, more solvent use, long processing time, and thermally degrading by-products. In contrast, modern and advanced extraction nonthermal technologies such as pulsed electric field (PEF) assist in easier and efficient identification, characterization, and analysis of bioactive ingredients. Other advantages of PEF include cost-efficacy, less time, and solvent consumption with improved yields. This review covers the applications of PEF to obtain bioactive components, essential oils, proteins, pectin, and other important materials from various parts of the plant. Numerous studies compiled in the current evaluation concluded PEF as the best solution to extract phytoconstituents used in the food and pharmaceutical industries. PEF-assisted extraction leads to a higher yield, utilizes less solvents and energy, and it saves a lot of time compared to traditional extraction methods. PEF extraction design should be safe and efficient enough to prevent the degradation of phytoconstituents and oils.

## 1. Introduction

Plants with complex structures contain phenolic compounds in cell vacuoles and a lipoprotein bilayer. However, the membrane envelope controls intracellular movement within the intact cells [1,2,3,4,5,6]. From medieval cultures, natural products and food have been extracted as an essential source of spiritual, cosmetic, and nutritious ingredients. The Pharaonic civilization (more than 4000 years ago) was the first to use solid–liquid extraction to separate aromas, colors, and other materials from plants [7]. Soxhlet, heat reflux, soaking, water percolation, maceration, magnetic stirrer, boiling, and grinding are the traditional practices used to obtain phytoconstituents but involve high solvent consumption, a long processing time, poor or low extraction yields, and few have thermal degradation risks. To overcome the limitations of these traditional technologies, numerous innovative non-thermal extraction techniques have been evaluated [8,9].

Pulsed electric field (PEF) has gained more attention in the past few years to extract beneficial materials from food waste/by-products through diffusion, osmosis, pressing, and drying [10]. It minimizes the deleterious effects of conventional heating processes [11,12,13]. PEF technology, as a promising alternative to other methods (boiling, microwave- and ultrasound-assisted extractions, etc.), has been used for the successful separation, intensification, stabilization, and dehydration of important compounds without affecting the nutritive properties [14,15,16,17]. Apart from improving the extraction, PEF has been recently proposed as a tool to induce stress in plant cells, thus stimulating the biosynthesis of active components [18].

Due to its ability to electroporate cell membrane, PEF is used as a pre-treatment to facilitate the recoveries of bioactive material followed by a subsequent traditional or novel extraction step [19]. The PEF method, when applied on water, showed a reduced temperature while consuming less solvent, and enhancing the rate at which the constituents were extracted [20]. PEF has also been used for recycling food waste and by-products through extracting valuable ingredients [21]. It decreased energy costs, improved the extraction yield, lessened the degradation of heat-sensitive substances, and purified extraction with no environmental impact [15]. The current review confines the latest research on PEF assisted extraction focusing on its types, mechanism, and applications in the food industry.

## 2. Working Principle of PEF-Assisted Extraction

**Figure 1:**
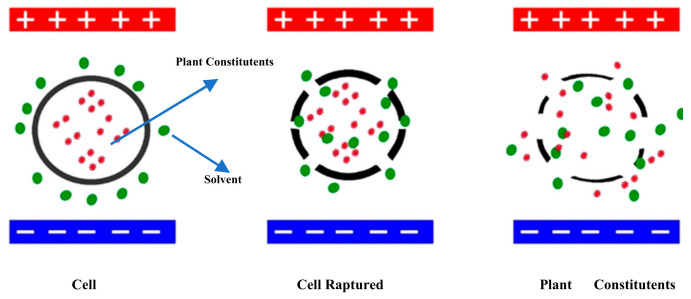
Electroporation mechanism for extraction.

The PEF technique uses moderate to high electric field strength (EFS) ranging from 100–300 V/cm in batch mode and 20–80 kV/cm in continuous mode extraction. Two views are common among various hypotheses concerning the potential PEF mechanism. In the biological cell membrane, one is the speeding of chemical-based reactions from various compounds to enhance the solubility of solvent [22] and the other is the electroporation process. Electro-permeabilization or electroporation involves an external electrical force, which enhances the permeability of cell membranes [23]. Food or any other targeted materials are placed between the electrodes and a high-voltage electric field. The cell membrane is punctured by creating hydrophilic pores, which opened protein channels. The sample experiences a force per unit charge called the electric field when high-voltage electrical pulses are applied through the electrodes. The membrane loses its structural functionality, and the plant material is extracted [24]. This mechanism has been shown in Figure 1.

An electric field can be applied either in exponentially decaying, oscillatory square waves, unipolar triangular, or bipolar pulses. The electroporation that occurs is either reversible or irreversible, but depending on the application, this effect can be controlled [25]. Generally, low specific energy (1–10 kJ/kg) and the time (nanoseconds to milliseconds) of the pulses cycle are efficient during the extraction process [26]. The electroporation of eukaryotic and prokaryotic cells and the formation of irreversible (permanent) and reversible (temporary) pores in their cell membranes were observed during the extraction process [27]. Irreversible electroporation increases the extraction process. However, EFS increases the cell membrane permeability, which depends on the size of the cell and cell geometry [28]. The strength of an electric field ranging from 0.1 to 10 kV/cm is enough for delicate plant tissues (e.g., pericarp or mesocarp of few fruits); however, tough materials such as seeds need high intensities (i.e., 10 to 20 kV/cm) for efficient extraction [29]. This also provides some additional benefits such as preserving the nutritional, and sensory characteristics of liquid foods [30].

The cells subjected to an external electric field exhibited a high transmembrane potential due to the accumulation of charge on the surface of the membrane. To protect the membrane, a cell has an electrical endurance limit, i.e., cell membranes can endure a specific electrical field strength without considerable damage. The critical electric field is the smallest threshold value for the same cell type. This will facilitate the development of pores in vulnerable areas of the membrane that can be reversible or permanent, depending on the strength of the electric field. The EFS is directly proportional to the magnitude of the damage. Damage is reversible (temporary) when the intensity is low to moderate (not much greater than the critical value). An electric field of high intensity caused irreversible (permanent) damage to the cell membrane [31]. Accordingly, PEF enhances the mass transfer by demolishing the structure of the cell membrane [22]. The increased membrane permeability led to cell breakdown, while the increased rate of mass transfer assisted the discharge of intracellular substances [32]. The degree of electroporation depends on the EFS, type and number of pulses waveform, treatment time, and targeted plant material [33]. However, the specific EFS depends on the geometry and distance of the electrodes.

## 3. PEF-Assisted Extraction Equipment

A PEF unit consists of a high-voltage pulse generator, a treatment chamber with a fluid managing assembly and a monitoring and control system [34]. Additionally, PEF equipment has a charger (to convert AC in DC) and a device that stores energy in the generator. A high-voltage circuit is switched on and off to generate electric pulses. During the discharge of high electric energy, the high-voltage value and short time pulses make this process more complicated, while the capacitor is continuously monitored and stepped up if the voltage is interrupted [35]. The treatment chamber consists of two separate electrodes (one electrode attached to the high-voltage generator and the other to the ground) and a gap; filled with the targeted food. The different electric potential on either side of the membrane generates an electric field, which depends on the electrodes type and the distance among them and with the sample. Other factors include the electric pulses nature, the configuration of the treatment chamber, and the product’s conductivity [28]. The PEF treatment parameters required to enhance polyphenols extraction, based on empirical experience, are categorized as high (E > 1 kV cm^−1^), medium (E ≈ 0.1–1 kV cm^−1^), and low (E < 0.1 kV cm^−1^) electric fields [35]. Generally, the PEF extraction process can be grouped into the following two broad categories: one is batch, and the other is a continuous system of extraction concerning the way of operation [22].

### 3.1. PEF Batch Extraction

A standard batch extraction unit comprises a pretreatment chamber for PEF and essential equipment for solid–liquid extraction. The pretreatment unit of PEF contains a cylindrical vessel of polypropylene (30 mm diameter on the inner side), having two stainless steel electrodes arranged parallel to each other at a 10-millimeter distance. The processing parameters include electrical field strength, pulses width, shape, number, and frequency. Inside the cylindrical unit, the sample with a little solvent was first treated between two electrodes and connected to a PEF generator. The treated sample was removed from the cylindrical unit and stirred at different velocities using a magnetic agitator to prevent solution evaporation [22].

**Figure 2:**
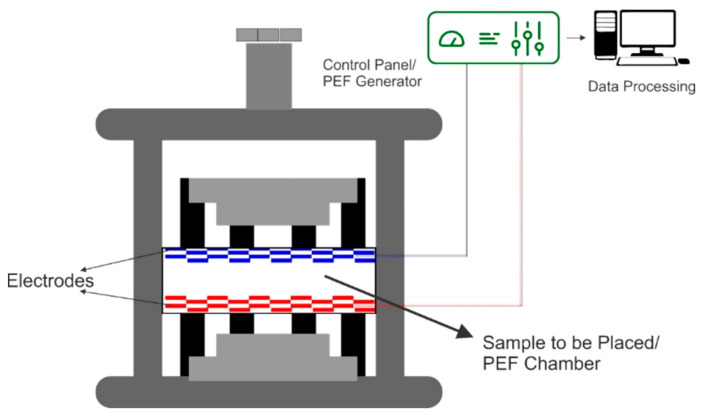
PEF (pulsed electric field) batch extraction system.

Using a PEF batch extraction method, various intracellular compounds such as lipid [36], red beetroot pigment [37], anthocyanin [35], betanine [22], oil [38], polyphenols [31], and cellulose [39] have been extracted. Figure 2 shows the PEF batch extraction system.

### 3.2. PEF Continuous Extraction 

The batch extraction process of PEF gave promising results in terms of extraction; however, a significant increase in the operating time has been observed, which is due to the low capacity of batch mode systems. Therefore, it is crucial to devise a PEF extraction process to perform it in regular conditions at the industrial level. Since then, the PEF continuous extraction method has been in great focus. The continuous-flow treatment chamber was first successfully used by Yongguang et al. [35] for the extraction of polysaccharides from *Rana temporaria chensinensis David* in 2006 [40]. Their results depicted that the extraction yield was 55.59% with PEF (20 kV/cm, 0.5% of KOH) as compared to the traditional extraction technique. In the past few years, the continuous extraction PEF applied successfully in laboratories to obtain fishbone, broccoli juice, eggshell, tomato juice, etc. This technology has been applied in industries thus far.

**Figure 3:**
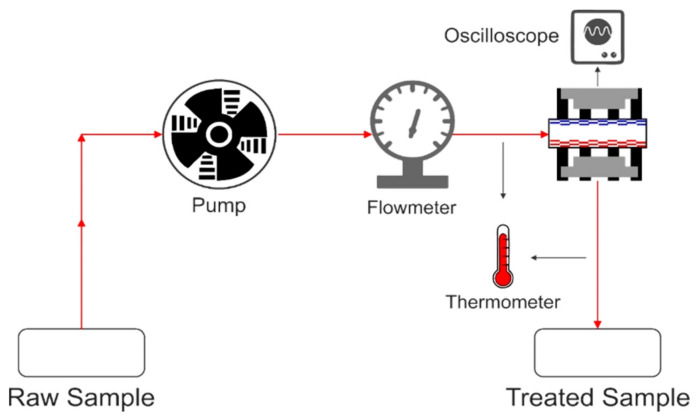
PEF (pulsed electric field) continuous extraction system.

A typical PEF continuous extraction system consists of a high-voltage pulse generator, a treatment chamber, a suitable product handling system, and a range of monitoring and controlling equipment. The oscilloscope can read the output voltage (up to 40 kV pulse voltage) directly, and the frequency is adjustable (40–3000 Hz). During continuous extraction PEF, the solvent mixture is pumped into the treatment chamber by a peristaltic pump at a constant fluid velocity. The cooling coil has a temperature of 25 °C in a water bath controlled by a thermostat during the extraction procedure. Coaxial and the cofield continuous PEF treatment chambers are currently widely used due to their simple configurations [22]. Figure 3 shows the PEF continuous extraction system.

## 4. Factors Influencing the PEF Extraction

The efficacy of the PEF extracting system depends not only on the processing parameters, but also on the solvent nature, sample composition (size, shape, pH, conductivity, etc.), and the extracted component’s size and position in the cell cytoplasm or vacuoles. Moreover, the characteristics of the tissues and cells have a powerful impact on the efficiency of PEF-assisted extraction [28,41]. The extraction process improved in the low ionic strength. The ionic strength affects the cytoplasmic system in terms of the cell’s compression and electroporation. However, the conductivity of the matrix affects the behavior of the electric field that passes through that matrix significantly [42].

EFS is a significant parameter in determining the degree of extraction as it affects the physical properties of the targeted compound such as diffusivity, surface tension, viscosity, and solubility [14]. The critical point is to assure the uniform distribution of electric fields across the treatment chamber. Electric field energy delivers through bipolar, exponential decaying, oscillatory, and square pulses. Among them, exponential square wave pulses are widely used in the PEF extraction process due to their high energy and lethal performance. Moreover, square waves are the most prevalent waveform used for the extraction process.

Generally, 12 to 45 kV/cm is enough to extract the valuable components from food; however, the PEF intensity depends on the food characteristics. Similarly, the extraction of target compounds is improved with an increase in the EFS due to high energy transfer in the food sample. Moreover, the PEF intensity (5–10 kV/cm) increased the hydrolysis rate and the total amino nitrogen content of abalone viscera protein. The results showed that the mild PEF intensity caused the cell membrane to undergo reversible electroporation. Hence, mild PEF intensity strength reclosed the cell electric hole. However, a PEF intensity greater than 10 kV/cm exudated the intracellular material due to an irreversible breakdown of the cell membrane. According to the authors, 20 kV/cm was the optimal EFS for a better extraction of bioactive compounds [43]. Lin et al. [44] found a significant increase (5.042 ± 0.04 to 6.996 ± 0.03 mg/mL) in the calcium malate extraction from an eggshell using PEF at 0 to 10 kV/cm EFS. According to the authors, the PEF treatments accelerated the movements between ionic groups and electrons having more kinetic energy, which increased the extraction rate of calcium malate and malic acid. However, the higher EFS facilitated the dissolution of calcium ions. Alternatively, when the pulse time raised from 16 to 20 s, the content of dissoluble calcium malate was significantly reduced. They further explained that the narrow pulses excited the material to resonance vibration. It was also established that, the extreme high EFS, i.e., 35 kV/cm, caused the vitamin C degradation of broccoli juice [14].

Treatment temperature is another important factor affecting the PEF extraction process [45]. PEF extraction technology is a nonthermal process, thus it operates in room or near room temperature. Higher temperatures usually decrease the viscosity of liquid solvents, which is destructive for the extraction process. Treatment time (pulse numbers and width) is another parameter to measure PEF efficiency [42]. However, an increase in treatment time could raise the temperature of the product. According to the results, longer pulses maximized the extraction rate of polyphenols from fresh tea leaves at 0.9 kV/cm for 0.5 s and 1.1 kV/cm for 3 s [46]. Other than that, the selection of solvent is also crucial for better PEF extraction. It involves numerous factors such as the solubility, conductivity, and polarity of the solvent. The increased solvent conductivity enhanced the cell membrane electroporation that ultimately improved the extraction rate. Similarly, the high solubility of the extract in the solvent and the strong polarity of the solvent increased the mass transfer rate and extraction rate [47,48,49].

## 5. Applications

### 5.1. Fruits and Vegetables

Due to their nutritional value worldwide and the fact that they are rich sources of beneficial antioxidants, minerals, vitamins and fibers [50], fruits, and vegetables are the most common source of nutrition. A variety of antioxidant compounds can be present in fruits and vegetables, including phenolics, carotenoids, anthocyanins, and tocopherols [51]. PEF is a promising method to extract bioactive compounds (anthocyanins, betanines, carotenoids, etc.) from fruit and vegetables, as shown in Table 1. Several factors depend on the extraction of bioactive compounds, such as the process of extraction, the solvent used for extraction and raw materials [52]. Traditional extraction techniques such as Hydro distillation, maceration, and Soxhlet require agitation, high temperatures, and chemical or organic solvents [53]. Nevertheless, the Soxhlet method needs considerable time for extraction and significant quantities of the solvent [54].

Leong et al. [55] evaluated the extraction of anthocyanins from grape juices using PEF technology with 20 µS pulse, 50 Hz frequency and 1.5 kV/cm electric field power. PEF-assisted extraction improved the release of vitamin C and anthocyanins as well as enhanced the antioxidant activity of grape juice as compared to untreated samples. The resultant extract had good phytochemical composition and protected the cells from oxidation [55]. Similarly, the PEF treatment enhanced the antioxidant activity (1.03 times) and extraction of polyphenols (1.44 times) of green grape juice [56]. Moreover, the PEF pretreatment enhanced the recovery of red raspberry (*Rubus idaeus* L.) juice by 9–25% without affecting the total phenolic and anthocyanin content. Furthermore, it enhanced the red raspberry press-cake extract that involved a 20% increase in phenolic content and a 26% increase in anthocyanin content [57]. In another study, PEF (13.3 kV/cm, 0–564 kJ/kg) enhanced the extraction yields of fermented grape (*Dunkelfelder*) pomace up to 22 and 55%. The results showed more recovery of anthocyanins as compared to traditional grinding methods, ultrasound-assisted extraction, and high-voltage electrical discharge [58].

PEF treatment is the most suitable nonthermal technique to extract phenols and flavonoids from onions without significant quality losses. Plant tissues contain a cytomembrane, which affects the movement of intracellular substances between cells. PEF treatments altered the functionality (permeability) of cytomembrane by disintegration and improved the movement of mass through cells, providing greater yields. Compared to the control samples, the PEF treatment significantly enhanced the phenolic compounds (102.86 mg GAE/100 g) by 2.2 times and the flavonoid compounds (37.58 mg QE/100 g) by 2.7 times in onions [59]. According to Fincan [60], the disintegration index was 0.86 ± 0.02 at 99 pulses of 3 kV/cm (4102 ± 239 J/kg) in the PEF-treated sesame seed cake. The extraction yields (total phenolic, antioxidant power, and antioxidant activity) produced using microwave and heat technology was comparable with PEF-assisted extraction [60]. Further, Sarkis et al. [61] stated that the disintegration index and the proteins and polyphenols content increased until 83 kJ/kg energy inputs in the case of spearmint [61].

Xue et al. [62] showed that PEF treatment (38.4 kV/cm, 272 μs) produced higher extraction yields of polysaccharides (97.7%), protein (48.9%), and polyphenolic compounds (50.9%) in white button mushrooms compared to traditional thermal treatment (95 °C for 1 h). The traditional technique has a prolonged treatment cycle as compared to the PEF system. However, PEF exhibited a fluid residence time of less than 2.6 min [62]. In the same context, Jeya et al. [25] investigated that PEF at 7 kV/cm and 2.5 kJ/kg resulted in a 4.2-fold enhancement in the yield of betanine as compared to untreated samples. According to the authors, 90% of betanine extraction was achieved in 35 min. Frontuto et al. [63] indicated that the PEF pretreatment of potato peel tissues greatly enhanced the extraction of phenolics. Accordingly, PEF pretreatment achieved the same amount (1062 mg GAE/kg) of phenolics in 144 min as compared to untreated samples (240 min) [63]. Similarly, PEF treatment (2 kV/cm, 11.225 kJ/kg) significantly enhanced the yield (13.3%) of phenolic content in olive paste [64].

**Table 1:** PEF Assisted extraction of Bioactive compounds.

Ref.	Raw Material	Extraction Technique	Pretreatment Condition	Extraction Condition	Solid-Solvent Ratio	Solvent	Yield	TPC	DPPH	FRAP	IC50	TFC	TAC	TCC
[65]	Cinnamon (*Cinnamomum verum*) powder	PEF-assisted extraction	Frequency: 1 Hz Voltage: 2–6 5.12 kV/cm No. of pulses: 40–60 NR	Temperature: ambient Time: 48 h	1:10 *w/v*	Ethanol 100 mL	5.06 %	505.9 mg GA/kg	91.7%	NR	NR	NR	NR	NR
[66]	*Nepeta binaludensis*	PEF-assisted extraction	No. of pulses:60 Frequency: 1 Hz Voltage: 6 kV No. of pulses: 60	Temperature: ambient Time: 48 h	1:10 *w/v*	Ethanol 100 mL	11.36%	417.85 mg GA/g	74.8%	1688.53 µmol Fe^2+^/g	0.32 mg/mL	NR	NR	NR
[24]	Thinned peach (*Prunus persica*)	PEF-assisted extraction	EFS: 0 kV/cm Specific energy: 0.61–9.98 kJ/kg Pulse frequency: 1 Hz No. of pulses per time: 30–150 µs	Temperature: 35 °C Time: 10 h	NR	Methanol 80% 200 mL water–methanol solvent	NR	83.3 mg GAE/100 g	57.8 %	NR	NR	54.3 CE/100 g	NR	NR
[67]	Tomato (*Solanum lycopersicum*) peel	PEF-assisted extraction	EFS:5 kV/cm Total specific energy: 5 kJ/kg	-	1:40 g/mL	Acetone	NR	NR	4.2 ± 0.4 mmol TE/100 g FW	NR	NR	NR	NR	80.4 ± 2.2 mg/100 g FW
			-	Temperature: 50 Time: 4 h Speed: 160 rpm					5.2 ± 0.4 mmolTE/100 g FW					84.0 ± 8.3 mg/100 g FW
[68]	Sweet Cherries (*Prunus avium*)	PEF-assisted pressing	Variable field strength: 1 kV/cm Frequency: 5 Hz Pulse width: 20 µs Total specific energy input: 10 kJ/kg	Pressure: 1.64 bar Time: 5 min	NR	NR	Juice yield40%	NR	NR	27.4%	NR	NR	29.2 ± 1.1 mg/100 mL	NR
	Sweet cherries (*Prunus avium*) press cake	PEF-assisted extraction	Variable field strength: 0.5–1 kV/cm Frequency: 5 Hz Pulse width: 20 µs Total specific energy input: 10 kJ/kg	Time: 24 h Temperature: 25 °C	5:1 mL/g Solvent–cake ratio	Acidified aqueous ethanol (50% ethanol; 0.5%HCl, *v/v*)	NR	NR	NR	21.0%	NR	NR	218.0 ± 14.8 mg/100 mL	NR
[15]	Moringa olifera dry leaves	PEF-assisted extraction	EFS: 7 kV/cm	Time: 40 min Temperature: ambient Pulse duration: 20 ms Pulse interval: 100 µs	NR	NR	NR	40.24 mg GAE/g of dry matter	98.31	108.22 µmoL AAE/g dry matter	NR	NR	NR	NR
[63]	Potato (*Solanum tuberosum*) peels	PEF-assisted extraction	Pulse width:3–25 µs Frequency: 1–450 Hz Electric field: 1 kV/cmSpecific energy: 5 kJ/kg	Time: 30–240 min Temperature: 20–50 °C Speed: 160 rpm	1:20 g/mL	Water–ethanol mixture Ethanol concentration 50%	NR	1263.5 ± 43 mgGAE/kg FW PP	877.17/kg FWPP	NR	NR	NR	NR	NR
[69]	Date palm (*Phoenix dactylifera*)	PEF-assisted ethanolic extraction	EFS: 3 kV/cm Frequenc:10Hz	Time: 6 h	4:1 *v/v*	Ethanol–water 300 mL	NR	67.35 mg GAE/100 g	50–72	NR	110 µL/mL	6.75 mg CE/100 g	2.08 mg/L	6.10 µg/mL
[70]	Tropical almond red leaves (*Terminalia catappa*)	PEF-assisted extraction	NR	Frequency: 1 Hz Electric field intensity: 0.75 kV/cm No. of pulses: 50 n	1:10	Water	74.6%	241.40 ± 2.15 mg GAE/g	93.40 ± 1.23%	NR	42 mg/mL	NR	NR	NR
[59]	Red onion (*Allium cepa*)	PEF-assisted water extraction	Pulse wide:100 µs Frequency: 1 Hz Electric field intensity: 2.5 kV/cm No. of pulse: 90 Specific energy: 0.23–9.38 kJ/kg	Time: 2 h Shaking speed: 200 rpm/min Temperature: 42.5 °C	NR	Distilled water 50 mL	NR	102.86 mg GAE/100 g FW	262.39 %	NR	NR	37.58 mg QE/100 gFW	NR	NR
[71]	Wild blueberries (*Vaccinium myrtillus)*	PEF-assisted pressing	EFS:3 kV/cm Total specific energy: 10 kJ/kg Frequency: 10 Hz Pulse width: 20 µs	Time: 8 min Pressure: 1.32 bar	NR	NR	56.3	45.5%	NR	35.9 %	NR	NR	77.5%	NR
[72]	Sour cherries (*Prunus cerasus*)	PEF-assisted pressing	EFS: 5 kV/cm total specific energy input:10 kJ/kg constant frequency: 10 Hz pulse width:20 µs	Pressing time: 9 min	NR	NR	37.7 g 100/g1	133.90 ± 2.67 mg 100/mL Juice	NR	6.60 ± 0.13 μmol TE/mL	NR	NR	53.30 ± 0.97 mg 100/mL Juice	NR
[73]	Fresh pomelo fruits (Shantian Variety)	-	Number of pulses of 30 Electric field intensity: 4 kV/cm	Temperature: 40 °C	40:60, *v*/*v*	90 mL ethanol–water	16.19 mg/mL (naringin) increased by about 20%	NR	38.58% increased by 70%	NR	NR	NR	NR	NR
[74]	Thawed blueberries	PEF-assisted pressing	EFSs: 1, 3 kV/cm Total specific energy: 10 kJ/kg Constant frequency: 20 Hz Pulse width: 20	Constant pressure: 1.32 bar Time: 8 min	NR	NR	36.3 ± 1%	8.0%	NR	NR	NR	NR	8.3%	NR

TPC: Total phenolic content; TFC: Total Flavonoid Content; TCC: Total carotenoid content; DPPH: Diphenyl picrylhydrazyl; FRAP: Ferric-reducing antioxidant power; TAC: Total anthocyanin content; IC50: half-maximal inhibitory concentration; FW: Fresh Water; FWPP: Fresh Weight Potato Peel; NR: Not reported.

### 5.2. Agro-Industrial Waste

Fruit and vegetable flesh or pulp is consumed while the other components, which are not mostly consumed, such as peel and seeds, contain a significant amount of various vital nutrients and phytochemicals [9,75,76]. For example, lemon, grapes, orange peels, avocado, jackfruit, longan, and mango seeds contain more than a 15% phenolic concentration, which is higher than those contained in fruit pulp [77]. In the food industry, much of the waste being produced is marked by the demand for biological oxygen and the demand for chemical oxygen. The food waste is rich in numerous bioactive components such as phenolic acids, flavonoids (hesperetin, quercetin, genistein, and kaempferol), and carotenoids (lutein and zeaxanthin); hence, it is concerned with the benefits of using PEF to recover valuable compounds that would contribute to the notion of zero waste.

Conventionally, these compounds are extracted by different treatments such as nanoemulsions used in the nutraceutical, cosmetics, and pharmaceutical industries [75]. The traditional solvent extraction techniques are time and energy consuming. For instance, the industrial batch extraction of polyphenols from grape peels is usually carried out at 50–60 °C for 20 h. Furthermore, the bioactive is enclosed in plant cell vacuoles and membrane bilayers (insoluble structures) that are not open to solvents. The usage of high temperature to enhance mass transfer and reduction in time may have serious drawbacks since high temperature (greater than 70 °C) caused rapid degradation of heat-sensitive components such as anthocyanins [78]. Kantar et al. [79] compared the total polyphenolic content of orange, grapefruit, and lemon in both juice extract and peel (flavedo/albedo) using PEF (3–10 kV/cm) as a pretreatment followed by the traditional extraction procedure (50% ethanol for 1h). The results showed an improved flavonoid content and total phenolic contents (2200 mg GAE/100 g) of fruit peels at 10 kV/cm [79].

### 5.3. Herbs and Spices

Herbs and spices have been widely used to strengthen or enhance the taste and preserve the quality of food along with their beneficial effects on human health. The plant essential oils contain 85% of polyphenols, terpenes, monoterpenes, and sesquiterpenes. However, essential oils (derived from herbs and spices) have 70 or more different molecules of phytochemicals such as terpenoids, polyphenols, flavonols, flavonoids, and tannins [80,81,82]. Spices have been used in traditional medicine since ancient times; however, their beneficial health effects have been experimentally recognized only in the last three decades. Recently, the antioxidant properties of black pepper (piperine), red pepper (capsaicin), turmeric (curcumin), fenugreek, ginger (gingero), garlic, clove (eugenol), and onion (quercetin) have been investigated by [83]. The antioxidants derived from spices have been evaluated for the prevention of various health-related disorders including atherogenesis.

The most common method used for extracting bioactive or essential oils from plant sources is Soxhlet extraction (hydro and steam distillation), while other traditional extraction techniques include maceration, engraving, and cohobation. However, steam distillation has been used widely for the commercial production of essential oils [84]. The traditional methods are complex multistage processes, which consumed more organic solvent, time, and energy while resulting in the loss of analytes. These factors are leading to the low selectivity of conventional extraction methods. In contrast, the modern PEF technique enhanced the extraction capacity of bioactive from metabolically active tissues via increasing osmotic dehydration with less energy input and an enhanced recovery of nutrients [85]. Furthermore, PEF extraction was facilitated by solvent diffusion and freeze-drying [11].

*Phyllanthus emblica* L. (*Syn. Emblica officinalis*) is an ancient herb well known for its functional properties. PEF (18 to 24 kV/cm, 300 to 1000 µs) treated Emblica juice showed an improved (9 times) extraction of quercetin and ellagic acid than thermally processed juice. According to the authors, 22 kV/cm was the optimum electric field power for 0.79 disintegration index within 500 µs [85]. Similarly, PEF extraction at 20 kV/cm using 70% ethanol and water increased the yield (12.69 mg/g) of ginsenosides as compared to ultrasound assisted extraction, microwave assisted extraction, heat reflux extraction, and pressurized liquid extraction. The authors stated that it took less than 1 s to complete PEF extraction, which is much lower than the other methods tested [86].

### 5.4. Leaves

According to Segovia et al. [87], PEF treatment (300 Hz, 30 kV) increased the polyphenols from 1.3 to 6.6% and the Oxygen Radical Absorption Capacity from 2.0 to 13.7% in *Borago officinalis* L. leaves. Moreover, PEF-assisted extraction increased the antioxidant capacity of the extracts and reduced the extraction times [87]. Barba et al. [88] established that PEF extraction treatments (0 to 141 kJ/kg, using water as a solvent) disrupt the plant cells of *Stevia rebaudiana* Bertoni leaves. PEF extraction improved conductivity (≈25%) and soluble matter extraction yields (≈33%) as compared to diffusion. Furthermore, PEF enhanced the antioxidant activity (50%), total phenolic compounds activity (80%), chlorogenic acid (93%), caffeic acids (55%), ferulic acid (90%), and protocatechuic acids (45%) [88]. Table 2 summarizes the range of parameters used in PEF-assisted extraction for the release of bioactive components from Agro-Industrial waste.

### 5.5. Oleaginous Seeds

The major oil crops include ground nuts, olive, linseed, cotton, hemp, castor, cottonseed, safflower, sesame oils, etc. Among them soy, rape, palm, and sunflower are the most significant oil-bearing plants [92]. Fats and oils are the most consumed items and are of much importance because of triacylglycerols [93]. Furthermore, edible oils are a major source of energy (calories) and essential vitamins. Moreover, oilseeds are placed second after grains as food reserves [94,95]. Pressing is a conventional method to extract oil from seeds, which squeezes the oil out of solid material (having more than 30% oil). After pressing, the press-cake is then subjected to solvent extraction. Traditionally, cold and hot-pressing technologies are used for the extraction of oil such as solvent extraction (Soxhlet) for flaxseed. On an industrial scale, generally, the solvent extraction step is applied by using a lot of hexane in the countercurrent extractors [96]. In contrast, PEF treatments (50 kV) were used to extract oil (including tocopherols, antioxidants, herbal cholesterol (phytosterols), and other functional compounds) from oleaginous material [15]. For instance, PEF (2 kV/cm, 11.25 kJ/kg, 25 Hz) using monopolar exponential decay pulses (0.3 µs) increased the oil yield (22.66 kg/100 kg) from olive paste as compared to the control sample (20.00 kg/100 kg) [97]. Table 3 illustrates that PEF-assisted extraction is a progressing nonthermal technique to increase the extraction yield of seed oils. PEF-assisted extraction assures higher extraction yields without a detrimental impact on the nutritional value and freshness of the product [98].

### 5.6. Microorganisms

Microorganisms such as bacteria, yeast, and algae are good sources for highly valued compounds such as enzymes, pigments, and nutrients as shown in Table 4. The major portion of such compounds resides inside the cell; therefore, it is essential to extract and purify them before use [103]. Microorganisms can carry out a broad range of reactions and are adaptable to a variety of conditions. They can be moved from nature into the laboratory to produce beneficial compounds on cheap sources such as carbon and nitrogen. Due to the biological activity of secondary metabolites originating from microbes, they are greatly beneficial for our nutrition and health. Research is progressed in the screening of naturally occurring microbial products, for the development of novel therapeutic agents. The search of novel chemicals is an important way forward to study the capacity of lesser-known or novel bacterial taxa [104].

Martínez et al. [105] used PEF (15–25 kV/cm, 60–150 μs, 10–40 °C) to enhance the extraction of a selective protein called phycocyanin (a water-soluble protein) from Artrosphira platensisto’s fresh biomass. According to the authors, low molecular weight compounds move directly through the cytoplasmic membrane after electroporation, while high molecular weight compounds require that the pores produced by the PEF treatment expand over time. Hence, the resultant delay of 150 min was recorded during the extraction. [105]. Similarly, Jaeschke et al. [106] also succeeded in achieving a high protein and phycocyanin yield from *Arthospira platensis* after a PEF treatment of 40 kV/cm utilizing pulses of 1 μs (112 kJ/kg) [106]. Martínez et al. [107] extracted another water-soluble protein (β-phycoerythrin) from *Porphyridium cruentum* by applying PEF for 24 h. However, this water-soluble phycobiliprotein was unobservable in the untreated cells, even after extended periods of incubation. The results showed that the extraction of β-phycoerythrin involved not only the diffusion of the pigment through the cell membrane, but also the disassembly from the cell organization of the molecule. In this way, PEF released the *P. cruentum* organelles (hydrolytic enzymes) that broke the bonds between the pigment and other cell compounds; thus, the water- β-phycoerythrin complex diffused, carried by a concentration gradient, through the membrane. The enzymatic autolysis of microalgae caused by PEF must be further investigated for the industrial implementation of PEF extraction technology [108]. Table 4 further confines some of the literature on PEF that involves the extraction of other phytoconstituents.

**Table 4:** The extraction of other phytoconstituents.

**Phycocyanin**						
**References**	**Raw** **material**	**PEF** **treatment**	**Extraction** **method**	**Extraction conditions**	**Solvent**	**Yield**
[105]	*Arthrospira platensis*	EFS: 25 kV/cm Treatment time: 150 μs Temperature: 40 °C	PEF-assisted extraction	Temperature: 20 °C Time: 360 min	19 mL of distilled water	Extraction yield 151.94 ± 14.22 mg/g
**Total Amino Nitrogen Content (TANC)**						
**References**	**Raw material**	**PEF treatment**	**Extraction** **method**	**Extraction** **conditions**	**Solvent**	**Yield**
[43]	Fresh abalone (*Haliotis Discus Hannai Ino*) viscera	NR	PEF-assisted enzymatic extraction	EFS: 20 kV/cm treatment times: 600 s	NR	42.35%, 175.20 mg/100 mL
**Lipids**						
**References**	**Raw** **material**	**PEF treatment**	**Extraction method**	**Extraction conditions**	**Solvent**	**Extracted** **lipid**
[109]	fresh microalgae *Auxenochlorella* *protothecoides*	Flow rate: 0.1 mL/s Pulse duration 1 µs EFS: 4 MV/m Specific energy: 150 kJ/L	NR	Time: overnight incubation conditions: Temperature: 25 °C Time: 20 h	Hexane–ethanol blend	97%
**Pectin**						
**References**	**Raw material**	**extraction method**	**Extraction device**	**Extraction conditions**	**Powder–solvent ratio**	**Yield**
[110]	Raw jackfruit (*Artocarpus heterophyllus*)	Combination of PEF- and microwave-assisted extraction	PEF generator microwave reactor	PEF conditions: Time: 5 min, EFS: 10 kV/cm. Microwave conditions: Time: 10 min, Power level of 650 W/g	1:4	18.3%
**Cellulose**						
**References**	**Raw material**	**PEF treatment**	**Extraction** **method**	**Solvent**	**Yield**	
[39]	Mendong Fiber *(Fimbristylis globulosa)*	EFS: 1.3 kV/cm Frequency: 20 kHz Time 30 s	PEF-assisted alkali extraction	NaOH sol. (300 mL) 60% conc.	97.8%	
**Pigment**						
**References**	**Raw material**	**PEF treatment**	**Extraction method**	**Extraction conditions**	**Yield**	
[111]	Algae paste of *Nannochloropsis oceanica*	Constant flow rate: 20 mL/min Temperature: 25 °C Square wave: 5 μs EFS: 10 kV/cm Total specific energy inputs:100 kJ/kg	PEF-assisted supercritical CO_2_ extraction	Pressure: 8, 14, and 20 MPa Fixed temperature: 35 °C CO_2_/biomass ratio: 53.3 kgCO_2_/kg DW Holding time: 7 min	Total carotenes: 36% Total chlorophyll: 52%	

EFS: Electric field strength; NR: not reported.

## 6. Conclusions

PEF uses a moderate to high electric field, keeping low energy, low solvent, and less time along with higher extraction yields of different bioactive compounds from fruits, vegetables, herbs, spices, leaves, and their wastes. Furthermore, PEF-assisted extraction enhanced the oil release from oleaginous seeds in comparison to other conventional techniques. Industrially, PEF continuous extraction technology showed promising results in the extraction rate of phytoconstituents. The efficacy of the PEF extracting system depends not only on the processing parameters but also on the solvent nature and sample composition. The targeted physicochemical properties include size, shape, pH, conductivity, etc. However, the extracted component’s size and position in the cell cytoplasm or vacuoles are also important factors. It is worthy to note that PEF exhibited potential yields of highly valued items such as enzymes, pigments, and nutrients from microorganisms such as bacteria, yeast, and algae. In future, PEF processing is a promising method for pharmaceuticals, food processing, and bioengineering industries due to its energy-efficient nature.

## Figures and Tables

**Table 2:** The extraction of bioactive compounds assisted by PEF pretreatment.

Ref.	Raw Material	PEF-Pretreatment	Extraction Method	Extraction Conditions	Solid-Solvent Ratio	Solvent	Yield	TPC	AA	TAC	DPPH	FRAP	TFC
[89]	Thawed blackcurrant (*Ribes nigrum*)	EFS: 1318 kV/cm Pulses: 315	Cold pressing	Power: 150 W Velocity: 70 rpm Pressing time: 1.5 min	NR	NR	NR	3.8 ± 0.2 mg GA/g	1.88 ± 0.06 mg GA/g	NR	NR	NR	NR
[72]	Blueberry (*Vaccinium myrtillus*) press cake	EFS: 5 kV/cm	Solid–liquid extraction	Temperature: ambient Time: 24 h Shaking speed: 150 rpm	6:1 mL/g Solvent to press cake ratio	50% Ethanol; 0.5% HCl, *v*/*v*	NR	89.3%	NR	111%	NR	80%	NR
[90]	Fresh tea leaves (*Camellia sinensis*)	EFS: 1.00 kV/cm Pulses: 100 Energy: 22 kJ/kg Temperature: 1.5 °C	Organic solvent extraction	Time: 2 h Temperature: room Stirring speed: 250 rpm	1:1000 Biomass to solvent ratio	50% acetone/water (*w/w*) solution	NR	398 mg/L	NR	NR	NR	NR	NR
[72]	Sour cherry (*Prunus cerasus*) press cake	EFS: 5 kV/cm Specific energy input: 10 kJ/kg Frequency: 10 Hz Pulse width: 20 µs	Solvent extraction	Time: 24 h Temperature: ambient	10:1 *v/w* solvent to press cake	Acidified aqueous methanol 70% MeOH and 0.5% HCl, *v/v*	NR	407.90 ± 18.54 mg/100 g	NR	168.50 ± 3.99 mg/100 g	NR	56.10 ± 1.25 μmol TE/g	NR
[91]	Basil leaves (*Ocimum Americanum*)	EFS: 2–3 kV/cm Time: 1–2 min	Conventional method: maceration	Time: 3 h Temperature: room	NR	Distilled water 300 mL	33.15% ± 2.484%	115.203 ± 1.115 mg GAE/g extract	NR	NR	NR	NR	75.816 ± 0.723 mg QE/g
[19]	Rosmary (*Salvia rosmarinusby*)―product	Frequency: 10 Hz Pulse width: 30 µs Pulses: 167 EFS: 1.1 kV/cm Specific energy input 0.36/kg 24 g of 0.1% aqueous NaCl (1:1.4 *w/v*)	Ultrasound assisted	Power: 200 W Temperature: 40 °C Time: 12.48 min	(1: 20 *w/v*)	100 mL of 55.19% aqueous EtOH	NR	297 mg GAE/100 g FW	NR	NR	593 mg TE/100 g FW	NR	NR
	Thyme (*Thymus vulgaris*) by-product	Frequency: 10 Hz Pulse width: 30 µs Pulses:167 EFS: 1.1 kV/cm Specific energy input 0.46 kJ/kg 24 g of 0.1% aqueous NaCl 24 g of 0.1% aqueous NaCl (1:1.5 *w/v*)						460 mg GAE/100 g FW			570 mg TE/100 g FW		
[57]	Raspberry press cake	EFS: 1 kV/cm, 3 kV/cm Total specific energy: 1 kJ/kg, 6 kJ/kg Frequency: 20 Hz Pulse width: 20 µs	Solvent extraction	Temperature: ambient Time: 24 h Shaking speed: 150 rpm	Solvent to press cake 6:1 mL/g	50% ethanol, 0.5% HCl, *v/v*	NR	420.8 ± 39.33 mg GAE mg/100 g	NR	50.7 ± 3.38 mg/100 g	NR	24%	NR

AA: Antioxidant activity; FRAP: Ferric-reducing antioxidant power; TPC: Total phenolic content; TPC: Total phenolic content; TFC: Total flavonoid content; EFS: Electric field strength; NR: not reported.

**Table 3:** PEF assisted extraction of oils.

Ref.	Raw Material	PEF Pretreatment Conditions	Extraction Method	Extraction Equipment	Extraction Conditions	Solvent	Oil Yield
[99]	Sunflower seeds	EFS: 7.0 kV/cm Frequency: 0.5 Hz Solvent content: 50 wt.% Time: 90 s Pulse width: 30 µs	Solvent extraction	Shaker	Frequency: 400/min Time: 3 h Temperature: room	Bioethanol	55.9%
[100]	Damask rose flowers	EFS: 20 kV/cm Pulse number: 8	PEF-assisted Hydro distillation	Clevenger-type micro-apparatus	Distillation temperature: 100 °C Distillation time: 2 h	10% sodium chloride	yield of 0.105% with a 50% increase in essential oil
[101]	Sunflower seeds	EFS: 7 kV/cm Frequency: 1.5 Hz Treatment time: 30 s Pulse width: 30 µs	Solvent extraction	Shaker	Frequency: 400/min Time: 3 h Temperature: room	Hexane 40 mL (50 wt.%)	48.24%
[64]	Olive fruits (Arroniz variety)	NR	Pilot PEF-assisted extraction	Pilot PEF system	Electric fields: 2 kV/cm Frequency: 25 Hz flow rate: 520 kg/h Specific energy: 11.25 kJ/kg	NR	22.66 kg/100 kg
[102]	Olives (Nocellara del Belice variety)	EFS: 2 kV/cm Frequency: 25 Hz Pulse width: 50 µs Mass flow rate: 2300 kg/h Energy delivered per pulse: 210 J Specific energy: 7.83 kJ/kg	NR	Hammer crusher Malaxation machine	Malaxation time: 30 min Temperature: 27 °C	NR	85.5%

EFS: Electric field strength; NR: not reported.

## Data Availability

Not applicable.
